# Genome Sequence of *Campylobacter jejuni* strain 327, a strain isolated from a turkey slaughterhouse

**DOI:** 10.4056/sigs.1313504

**Published:** 2011-04-25

**Authors:** Monica Takamiya, Asli Ozen, Morten Rasmussen, Thomas Alter, Tom Gilbert, Dave W. Ussery, Susanne Knøchel

**Affiliations:** 1 Department of Food Science, Faculty of Life Sciences, University of Copenhagen, Rolighedsvej 30, DK-1958 Frederiksberg C, Denmark.; 2 Center for Biological Sequence Analysis, Department of Systems Biology, Technical University of Denmark, Kemitorvet, Building 208, DK-2800 Lyngby, Denmark.; 3 Center for GeoGenetics, Faculty of Science, University of Copenhagen, Ole Maaløes Vej 5, DK-2200 Copenhagen N, Denmark.; 4 Institute of Food Hygiene, Freie Universität Berlin, Königsweg 69, 14163 Berlin, Germany.

## Abstract

*Campylobacter* is one of the leading causes of food-borne gastroenteritis and has a high prevalence in poultry. *Campylobacter jejuni subsp. jejuni* 327 is a subspecies of the genus *Campylobacter* of the family *Campylobacteraceae* in the phylum *Proteobacteria*. The microaerophilic, spiral shaped, catalase positive bacterium obtains energy from the metabolism of amino acids and Krebs cycle intermediates. Strain 327 was isolated from a turkey slaughter production line and is considered environmentally sensitive to food processing (cold, heat, drying) and storage conditions. The 327 whole genome shotgun sequence of 1,618,613 bp long consists of 1,740 protein-coding genes, 46 tRNA genes and 3 rRNA operons. A protein based BLAST analysis places the turkey isolate 327 close to the human clinical strain 81116 (NCTC 11828).

## Introduction

*Campylobacter* is known worldwide as a common cause of human bacterial diarrhea; however, it is commensal in the gastrointestinal tract of many domestic and wild animals, especially birds. In 2008, campylobacteriosis remained the most frequently reported zoonotic disease in humans in the European Union with 190,566 confirmed cases [1]. Broiler meat and broiler flocks throughout the production chain in many EU-Member States, along with raw milk were reported as the most important food vehicles in food-borne *Campylobacter* outbreaks in 2008.

## Classification and features

**Figure 1:**
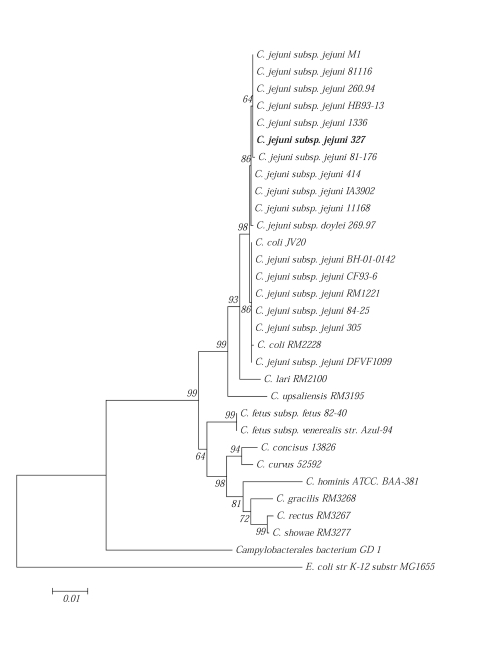
Phylogenetic tree based on 16S rRNA highlighting the position of *C. jejuni* 327 relative to the other type and non-type strains within the species *Campylobacter jejuni.* Strains shown are those within *Campylobacter jejuni* having corresponding NCBI genome project IDs listed below. The strains and their corresponding GenBank accession numbers (and, when applicable, draft sequence coordinates) for 16S rRNA genes are (type=^T^): *C. jejuni subsp. jejuni* NCTC 11168^T^, AL11168; *C. jejuni subsp. jejuni* M1, CP001900; *C. jejuni subsp. jejuni* 81116, CP000814; *C. jejuni subsp. jejuni* 260.94, AANK01000000*; C. jejuni subsp. jejuni* HB93-13, AANQ01000000; *C. jejuni subsp. jejuni* 1336, ADGL01000000; *C. jejuni subsp. jejuni* 327, ADHM00000000; *C. jejuni subsp. jejuni* 81-176, CP000538; *C. jejuni subsp. jejuni* 414, ADGM01000000; *C. jejuni subsp. jejuni* IA3902, CP001876; *C. jejuni subsp. doylei* 269.97, CP000768; *C. coli* JV20, AEER01000000; *C. jejuni subsp. jejuni* BH-01-0142, ABKD01000000; *C. jejuni subsp. jejuni* CF93-6, AANJ01000000; *C. jejuni subsp. jejuni* RM1221, CP000025; *C. jejuni subsp. jejuni* 84-25, AANT02000000; *C. jejuni subsp. jejuni* 305, ADHL00000000; *C. coli* RM2228, AAFL01000000; *C. jejuni subsp. jejuni* DFVF1099, ADHK00000000; *C. lari* RM2100, CP000932; *C. upsaliensis* RM3195, AAFJ01000000; *C. fetus subsp. fetus* 82-40, CP000487; *C. fetus subsp. venerealis* str. Azul-94, ACLG01000000; *C. concisus* 13826, CP000792; *C. curvus* 52592, CP000767; *C. hominis* ATCC. BAA-381, CP000776; *C. gracilis* RM3268, ACYG01000000; *C. rectus* RM3267, ACFU01000000; *C. showae* RM3277, ACVQ01000000; *Campylobacterales bacterium* GD 1, ABXD01000000; *E. coli* str K-12 substr MG1655, U00096. The tree uses sequences aligned by PRANK [23-25], which by default uses the Hasegawa, Kishino and Yano (HKY) model with empirical base frequencies and kappa=2. The tree is built with MEGA4 [26] using Neighbor-Joining method and 1000 re-samplings to calculate bootstrap values. *C. jejuni* 327 is found in the *C jejuni subsp. jejuni* cluster that has a 100% bootstrap value along with the *C. coli* species. *E. coli* K-12 was used as an outgroup.

The genus *Campylobacter* belongs to the *Epsilonproteobacteria* [2] and is classified in the family *Campylobacteraceae* [3, Table 1], which includes the genera *Campylobacter*, *Arcobacter, Dehalospirillum* and *Sulfurospirillum*. The closest genetically related genera are *Helicobacter* and *Wolinella*, which together belong to the family *Helicobacteraceae* [7,22]. Currently, available genomes of the genus *Campylobacter* comprises 29 species and 4 subspecies (see phylogenetic tree, Figure 1). The most commonly isolated pathogenic species are *C. jejuni*, *C. coli* and *C. fetus*. All these species have small genomes (1.6–2.0 megabases) and can establish long-term associations with their hosts, sometimes with pathogenic consequences.  Figure 1 shows the phylogenetic neighborhood of *C. jejuni* 327 in a 16S rRNA based tree.

**Table 1:** Classification and general features of *C. jejuni* 327 according to the MIGS recommendations [4]

**MIGS ID**	**Property**	**Term**	**Evidence code**
		Domain *Bacteria*	TAS [5]
		Phylum *Proteobacteria*	TAS [6]
		Class *Epsilonproteobacteria*	TAS [2,7,8]
	Current classification	Order *Campylobacterales*	TAS [2,7]
		Family *Campylobacteraceae*	TAS [3]
		Genus *Campylobacter*	TAS [3,9-15]
		Species *Campylobacter jejuni*	TAS [9,10]
		Strain 327	TAS [16]
	Gram stain	negative	TAS [9]
	Cell shape	Helical or curved rods can be observed in short or longer chains that can be V-, S-, or comma-shaped	TAS [9]
	Motility	motile via lateral flagella	TAS [9]
	Sporulation	Non-sporulating	TAS [9]
	Temperature range	thermophilic, 37-42°C	TAS [17]
	Optimum temperature	42°C	TAS [17]
	Salinity	sensitive to 1% NaCl	TAS [18]
MIGS-22	Oxygen requirement	microaerophilic (optimal concentrations of O_2_ [5–10%] and CO_2_ [3–5%])	TAS [9]
	Carbon source	peptides and amino acids	TAS [3]
	Energy source	amino acids and Krebs cycle intermediates for energy production	TAS [3]
MIGS-6	Habitat	Commensal organism in birds, primary enteric pathogen in humans	TAS [17]
MIGS-15	Biotic relationship	Free living	TAS [17]
MIGS-14	Pathogenicity	pathogenic	TAS [17]
	Biosafety level	2	TAS [4,19]
	Isolation	Poultry processing plant during slaughter of turkey batches Source of sampling: after killing	TAS [20]
MIGS-4	Geographic location	Different poultry processing plants located in Germany	TAS [20]
MIGS-5	Sample collection time	Autumn 2002	TAS [20]

Evidence codes - IDA: Inferred from Direct Assay (first time in publication); TAS: Traceable Author Statement (i.e., a direct report exists in the literature); NAS: Non-traceable Author Statement (i.e., not directly observed for the living, isolated sample, but based on a generally accepted property for the species, or anecdotal evidence). These evidence codes are from of the Gene Ontology project [21]. If the evidence code is IDA, then the property was directly observed for a live isolate by one of the authors, or an expert mentioned in the acknowledgements.

### Chemotaxonomy

All *Campylobacter* species contained menaquinone-6 (2-methyl-3-farnesyl-farnesyl-1,4-naphthoquinone) and methyl-substituted menaquinone-6 (2,[5 or 8]-dimethyl-3-farnesyl-farnesyl-1,4-napthoquinone) as the major isoprenoid quinones. The latter menaquinone has not been reported in other bacteria and may prove to be a useful chemical marker of *Campylobacter* species. *Campylobacter jejuni* and most strains of *Campylobacter coli* were distinguished from other *Campylobacter* species by the presence of a C_l9_ cyclopropane fatty acid acid in whole cell hydrolysates [21,27]

## Genome sequencing and annotation

### Genome project history

*Campylobacter jejuni* strain 327, one of the strains present in a turkey production line, was isolated from turkey skin surface swabs [20], and was selected for sequencing based on the sensitivity to environmental conditions in food-related environments [28]. Sequencing and finishing were performed by the Department of Biology (KU-NAT) and the Institute of Food Science (IFV) at the University of Copenhagen. The annotation was performed by the Institute for Genome Science (IGS, University of Maryland). The manual curation was completed by IFV and will be presented for public access with the publication of the Genome Announcement article. Genome analysis was performed by the Center for Biological Sequence Analysis (CBS) at the Technical University of Denmark (DTU). The Whole Genome Shotgun (WGS) project has been deposited at DDBJ/EMBL/GenBank under the project ID 41643 and accession number ADHM01000000. A summary of the project information is shown in Table 1 and Table 2 according to the Minimum Information about a Genomic Sequence (MIGS) recommendations [29].

**Table 2:** Genome sequencing project information

**MIGS ID**	**Property**	**Term**
MIGS-29	Sequencing platform	454 Life Sciences
MIGS-31	Finishing quality	Improved high-quality draft
MIGS-31.2	Fold coverage	20 ×
MIGS-32	Genome database release	with SIGS publication
	GenBank ID	41643
	Genbank Date of Release	with SIGS publication
	Project relevance	Food safety

### Growth conditions and DNA isolation

The turkey strain 327 was provided by Thomas Alter [20], and showed a sensitive phenotype to gentle food processing stresses [28]. *C. jejuni* cells were grown at 42 °C under microaerobic conditions (5% O_2,_ 10% CO_2_, 85% N_2_). Stocks were stored at -80°C in Brain Heart Infusion broth (BHI) (Oxoid CM225, England) supplemented with 15% glycerol. The frozen stocks were transferred to Blood Agar Base No.2 (Oxoid CM271, England) amended with 5% horse blood and incubated in a microaerobic atmosphere (5% O_2_, 10% CO_2_, 85% N_2_) at 42 °C for 24–72 h. The respective cultures were subsequently re-streaked on Blood Agar Base No.2 plates. After 24 hours of growth, a 3/4 loop-full of bacteria was resuspended in 1 ml phosphate buffered saline (PBS, Oxoid BR0014, England) and vortexed to ensure no bacterial clumps. Cells were centrifuged at 14,000 × g using a benchtop Sartorius centrifuge (model Sigma 1-14) and the medium was decanted. The cells were resuspended in 200 µl PBS for genomic DNA isolation using the Easy-DNA^TM^ Kit (Invitrogen, K1800-01). The protocol was followed as described by the manufacturer. A yield of approximately 10 mg of total genomic DNA was obtained for each *C. jejuni* strain.

### Genome sequencing and assembly

Pyrosequencing of *C. jejuni* strain 327 was performed on a Genome Sequencer GS FLX System (454 Life Sciences, Branford, CT, USA) at the Faculty of Biology, University of Copenhagen (KU-NAT). GS FLX sequencing was performed following the manufacturer’s protocol with minor modifications. Briefly, library preparations were done from 3μg of DNA using the shotgun library protocol with Multiplex Identifiers (MID) tags for each bacteria/sample, and DNA was released using heat instead of NaOH [30,31]. Libraries were quantified by qPCR as described in [32], and sequenced on a full GS FLX-LR70 plate. Genome sequences resulted in sequence reads which passed the length and quality criteria of the machine software. Draft assemblies were based on 134,679 total reads with 20-fold coverage of the genome. The 454 data files were loaded into the CLC Genomics Workbench version 3.7.1 (CLC Bio, Aarhus, Denmark). The initial reference was created using the human clinical strain 81116 [33] (NCTC 11828) as scaffold, yielding 133,175 matched reads (99% of match). For *de novo* assembly the 134,679 sequence reads were condensed to 48 contigs.

### Genome annotation

The *C. jejuni* genome sequences were automatically annotated using the Annotation Engine and the initial GenBank files were generated at the Institute for Genome Sciences (IGS, University of Maryland). These annotations and the GenBank files were further refined and corrected at the Center for Biological Sequence Analysis (CBS) at the Technical University of Denmark (DTU) by reference to codon usage, positional base preference methods and comparisons to the non-redundant protein databases using BLAST [34]. In-house Perl scripts from CBS and the Sequin program provided by NCBI [35] were used in this refinement process. The entire DNA sequence was also compared in all six potential reading frames against UniProt. Furthermore, the RNAmmer 1.2 server was used for ribosomal RNA predictions of 5S, 16S, and 23S [36]. The outcome of all these predictions was corrected on September 14th 2010.

## Genome properties

The *C. jejuni* 327 genome was found to be 1,618,613 bp long, and contains 1,740 protein coding genes as identified with the gene prediction program Prodigal version 1.20 [37], Table 3). The average G+C content is 30.4%, and there are 43 tRNAs and 5 rRNA genes found using the respective prediction server [36,40]. *C. jejuni* strain 327 does not contain any plasmids. Strain 327 contains 10 homopolymeric G tracts (HGTs, defined as tracts of >7 consecutive G-residues), fewer than the other complete genome sequences described to date (29 in NCTC 11168, 25 in RM1221 and 19 in 81-176 [41-43]). Variation in the length of homopolymeric G tracts may be produced by slipped-strand mispairing during replication [44], and can evolutionarily affect changes on the genome sequence. Thus, the number of hypervariable G tracts can give important hints on the genetic stability of the strain of *C. jejuni* studied. Of the 1,786 genes predicted, 1,740 were protein-coding genes, and 5 rRNA genes; 7 pseudogenes were identified. The majority of the protein-coding genes (97%) were assigned with a putative function while the remaining ones were annotated as hypothetical proteins. The distribution of genes into COGs functional categories is presented in Table 4.

**Table 3:** Genome Statistics

**Attribute**	**Value**	**% of Total**
Genome size (bp)	1,618,613	100.00%
DNA coding region (bp)	1,495,833	92.4%
DNA G+C content (bp)	492,058	30.4%
Number of replicons	1	
Extrachromosomal elements	0	
Total genes	1,786	100.00%
tRNA genes	46	2.58%
rRNA genes	5	0.28%
Protein-coding genes	1,740	97.42%
Pseudo genes	9	0.5%
Genes with function prediction	1,383	77.43%
Genes in paralog clusters	21	1.18%
Genes assigned to COGs	1,280	71.67%
Genes assigned Pfam domains *	1,350	75.59%
Genes with signal peptides [38]	263	14.7%
Genes with transmembrane helices	338	18.92%
CRISPR repeats [39]	1	

* E-value cutoff is 0.05.

**Table 4:** Number of genes associated with the general COG functional categories

**Code**	**value**	**%age**	**Description**
J	259	7.4	Translation, ribosomal structure and biogenesis
A	0	0	RNA processing and modification
K	89	2.6	Transcription
L	134	3.8	Replication, recombination and repair
B	0	0	Chromatin structure and dynamics
D	33	0.9	Cell cycle control, mitosis and meiosis
Y	0	0	Nuclear structure
V	42	1.2	Defense mechanisms
T	101	2.9	Signal transduction mechanisms
M	237	6.8	Cell wall/membrane biogenesis
N	114	3.3	Cell motility
Z	2	0.1	Cytoskeleton
W	0	0	Extracellular structures
U	102	2.9	Intracellular trafficking and secretion. and vesicular transport
O	153	4.4	Posttranslational modification. protein turnover. chaperones
C	206	5.9	Energy production and conversion
G	110	3.2	Carbohydrate transport and metabolism
E	303	8.7	Amino acid transport and metabolism
F	95	2.7	Nucleotide transport and metabolism
H	168	4.8	Coenzyme transport and metabolism
I	67	1.9	Lipid transport and metabolism
P	210	6	Inorganic ion transport and metabolism
Q	38	1.1	Secondary metabolites biosynthesis, transport and catabolism
R	336	9.7	General function prediction only
S	176	5.1	Function unknown
-	506	14.5	Not in COGs

## Genome Atlas construction

**Figure 2:**
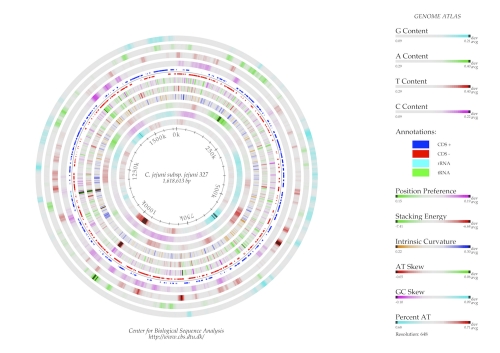
Genome Atlas of *C. jejuni* strain 327. The legend to the right explains what is represented from the outer to the inner circle. Shown are the fraction of each nucleotide along the genome (first four circles counting inwards), the coding sequences on the positive (clockwise strand), the AT and GC skew, and the percent AT.

The genome atlas of *C. jejuni subsp. jejuni* 327 was generated using the Genewiz program (Figure 2). In order to create the atlas, a FASTA file containing the nucleotide sequence in one piece and an annotation file showing the position of the genes were used. The FASTA file was created by concatenating the nucleotide sequences of the contigs. In the atlas, gene annotation, base content, AT and GC skew, percent AT and some structural properties of the DNA were shown. The structural properties are Position Preference, Stacking Energy and Intrinsic curvature which are all related to the flexibility and strength of the DNA molecule [45].

Available sequence data from completed strains (NCTC 11168, 81116 (NCTC 11828), RM1221, 81–176, 269.97, M1) and ongoing *C. jejuni* sequencing projects (strains 84–25, 260-94, HB93-13, CF93-6, CG8421, CG8486) obtained from the NCBI database at the time of writing, were used for homology searching of genes in selected loci using the program BLASTP [34]. It revealed a high protein sequence homology with strain 81116 (NCTC 11828), first isolated from a case of campylobacteriosis associated with a human waterborne outbreak [46]. The initial reference assembly using strain 81116 [33] (NCTC 11828) as scaffold created 133,175 matched reads (99% of match). In addition, *C. jejuni* 327 genome contains only a single *tonB* gene as compared to 2 or 3 genes in other *C. jejuni* strains, and lacks the ferric enterobactin uptake receptor CfrA and TonB-dependent outer membrane receptor for iron uptake [47]. Strain 327 also lacks the transcriptional regulator *mar* A (multiple antibiotic resistance) locus, first described for *E. coli* [48]. The *marA* locus mediates global stress response and affects the expression of iron-sulfur cluster proteins involved in sensing O_2_ and iron. The lack of this gene could explain the phenotype of strain 327 observed under some environmental stresses [28].
